# Enhancing human strength via neural modulation: mechanisms of maximal voluntary contraction and translational interventions

**DOI:** 10.3389/fphys.2025.1695665

**Published:** 2025-11-05

**Authors:** Yudai Takarada

**Affiliations:** Faculty of Sports Sciences, Waseda University, Saitama, Japan

**Keywords:** maximal voluntary contraction, MVC force, intracortical inhibition, descending drive, afferent feedback, neuroplasticity, primary motor cortex, M1

## Abstract

Maximal voluntary contraction (MVC) is a key determinant of human strength, mobility and functional performance. While muscle morphology contributes to MVC force, neural factors—particularly cortical and spinal excitability and inhibition—play a central role in motor unit recruitment. Despite its importance, the neurophysiological mechanisms regulating MVC remain underrepresented in literature. This narrative review synthesizes current evidence on the neural substrates of MVC, including intracortical inhibition, descending drive, afferent feedback, and neuroplasticity within the primary motor cortex (M1) and the corticospinal tract. A structured search of PubMed and Google Scholar identified studies examining both mechanisms and interventions. Intervention strategies were categorized into four domains: cognitive-behavioral techniques (e.g., verbal encouragement, unconscious goal priming), afferent-driven approaches, pharmacological modulation, and neuromodulatory stimulation. Studies were appraised for methodological rigor and translational relevance, highlighting the latent capacity of the motor system to exceed conventional MVC thresholds through targeted modulation of excitatory and inhibitory circuits. Evidence supports the efficacy of neuromodulatory and cognitive interventions in enhancing MVC force, particularly in older adults, athletes, and clinical populations. However, variability in protocols and outcome measures limits the comparability across studies. Further research is warranted to clarify the neurophysiological basis of MVC enhancement and to develop optimized, context-specific strategies for rehabilitation and performance.

## 1 Introduction

The muscular force generated during human maximal voluntary contraction (MVC) is primarily governed by both neural activity and muscle morphology. When morphological factors—such as muscle architecture (Lieber and Fridén, 2000, fiber-type composition, and muscle cross-sectional area (CSA) (Andersen, 2003)—are held constant, the ability to voluntarily recruit motor units becomes the principal determinant of MVC force. Notably, even under conditions of maximal voluntary effort, approximately half of individuals fail to achieve complete muscle-fiber activation due to limitations in neural drive (Belanger and McComas, 1981). This deficit has been attributed to insufficient supraspinal input targeting motor units (Belanger and McComas, 1981). Early work by Ikai and Steinhaus (1961) proposed that MVC force is constrained by psychological inhibitory factors under conventional conditions. Interestingly, self-generated shouting has been shown to transiently elevate motor system excitability and potentiate MVC force output (Takarada and Nozaki, 2021; Takarada and Nozaki, 2022a). These findings suggest that the motor system typically operates below its maximal capacity, retaining a latent potential for force production beyond conventional MVC thresholds (Takarada and Nozaki, 2014). Accordingly, this review aims to elucidate the key neural mechanisms underlying MVC force generation and examine how these mechanisms can be modulated to unlock untapped force capacity. Enhancing MVC force holds clinical and performance-related significance, with potential benefits for athletes, older adults, and patients undergoing rehabilitation.

MVC force is widely recognized as a reliable indicator of maximal strength in isometric muscle assessments (Berger, 1962). Typically, MVC is performed at a fixed joint angle against an immovable resistance connected to a transducer—such as strain gauges, cable tensiometers, or force platforms—that precisely quantify exerted force. Isometric assessments, including MVC, demonstrate high test-reliability across both single- and multi-joint protocols and are grounded in established principles of muscle physiology (Wilson and Murphy, 1996). These attributes make isometric MVC testing a valuable tool in both clinical and athletic contexts, enabling practitioners to design rehabilitation and training programs that enhance performance and mitigate injury risk.

Voluntary muscular force production is modulated by cortical and spinal excitability and inhibition, motor unit number and size, and discharge properties (Pion et al., 2017). Sustained maximal and submaximal contractions induce muscle fatigue, reflected by declines in voluntary activation (central fatigue), partially attributable to reduced output from the primary motor cortex (M1)—a phenomenon referred to as supraspinal fatigue (Smith et al., 2007). In contrast, brief maximal and submaximal contractions increase corticospinal inhibition, as evidenced by prolonged silent periods following transcranial magnetic stimulation (TMS) over the contralateral M1 (Yacyshyn et al., 2016). A meta-analysis revealed that strength training interventions substantially reduce short-interval intracortical inhibition and moderately shorten silent period duration (Kidgell et al., 2017), collectively indicating a reduction in cortical inhibitory tone. Intracortical circuits within M1 and the reticulospinal tract have been implicated in neural adaptations following 15 weeks of unilateral strength training in nonhuman primates, whereas changes in corticospinal and motoneuronal pathways were less pronounced or absent (Glover and Baker, 2020). Given the current limitations in directly assessing reticulospinal function in humans, it is reasonable to focus on M1 and corticospinal tract activity as key contributors to strength gains following resistance training. This perspective aligns with evidence indicating that neural adaptations are not primarily driven by intrinsic motoneuron properties, corticomotoneuronal synaptic efficacy, or descending transmission efficacy (Ansdell et al., 2020; Nuzzo et al., 2017). Thus, M1 and corticospinal tract activity constitute central components of the neural substrate governing human muscle force generation, with strength improvements linked to reduced intracortical inhibition and functional reorganizations within M1 circuits. A deeper understanding of these mechanisms may inform the development of optimized training protocols aimed at maximizing strength outcomes.

Importantly, MVC force can be augmented by several neural-based interventions, even under conditions of maximal voluntary effort. These include shouting (Takarada and Nozaki, 2021; Takarada and Nozaki, 2022a), caffeine ingestion (Behrens et al., 2015), verbal encouragement (Binboğa et al., 2013; Jung and Hallbeck, 2004; McNair et al., 1996), TMS (Pomeroy et al., 2007; Urbach et al., 2005), and electrical stimulation (Hortobágyi and Maffiuletti, 2011). For example, shouting-induced increases in MVC force are linked to heightened excitatory input to M1, thereby potentiating corticospinal tract activity (Takarada and Nozaki, 2021; Takarada and Nozaki, 2022a). These interventions and their underlying neural mechanisms will be discussed in Section 3. Enhancing MVC force may improve rehabilitation outcomes in patients with quadriceps weakness (Urbach et al., 2005), stroke survivors (Eng, 2004), and individuals with multiple sclerosis (Ramari et al., 2020). Moreover, healthy older adults may benefit from increased MVC force through improved gait stability, reduced fall risk, and enhanced quality of life (Marshall-McKenna et al., 2021). Furthermore, MVC augmentation may enhance athletic performance during both training and competition (Douglas et al., 2021; Takarada et al., 2002). These applications will be explored in detail in Section 4.

This review synthesizes recent advances in the neural basis of muscle force generation, examines the potential to modulate these neural mechanisms for enhanced muscle strength, discusses clinical and athletic applications, and outlines future research directions informed by the author’s expertise.

## 2 Literature search

To ensure a comprehensive and focused synthesis of relevant literature, a structured search was conducted in February 2025 using PubMed and Google Scholar, covering all years from database inception. The following keyword combinations were used: “neural mechanisms,” “neural basis,” “maximal muscle force,” “maximal voluntary contraction,” “MVC,” “manipulation,” “rehabilitation,” “older adults,” “elderly,” “sports,” “exercise,” “sports science,” and “stimulation.”

Search results were manually screened in three stages: title review, abstract evaluation, and full-text assessment. Inclusion criteria were: (1) studies addressing the neural regulation of maximal voluntary contraction or muscle force, (2) investigations of intervention strategies targeting neural mechanisms, and (3) relevance to clinical, aging, or athletic populations. Only peer-reviewed articles published in English were considered. Studies focusing solely on peripheral muscle adaptations without neural components were excluded.

Additional articles were identified through backward citation tracking of selected papers. This narrative review prioritizes mechanistic insights and translational relevance, rather than exhaustive meta-analytic synthesis.

## 3 Modulation of muscle force and underlying neural mechanisms

This section examines various approaches for enhancing MVC force and synthesizes current insights into the associated neurophysiological substrates. MVC force can be augmented through a wide range of interventions, encompassing behavioral (Takarada and Nozaki, 2021; Takarada and Nozaki, 2022a) and cognitive (Takarada and Nozaki, 2014; Takarada and Nozaki, 2024) strategies as well as electrical (Hortobágyi and Maffiuletti, 2011) and magnetic stimulation (Pomeroy et al., 2007). The appropriateness of each approach depends on its intended application and contextual demands (e.g., athletic performance vs. rehabilitation, acute vs. long-term MVC enhancement) (Krogh et al., 2022; Takarada and Nozaki, 2022b).

These interventions engage a diverse array of neural mechanisms, some of which are well-characterized, while others remain poorly understood. MVC enhancement has been demonstrated not only through self-generated shouting (Takarada and Nozaki, 2021) but also via subthreshold goal priming with motivational rewards (Takarada and Nozaki, 2014), both of which are hypothesized to increase excitability within the M1 and/or the corticospinal tract. Similar improvements in MVC force, accompanied by increased M1 and corticospinal excitability, have been reported following repetitive transcranial magnetic stimulation (rTMS) targeting the contralesional M1 during the early post-stroke phase (Pomeroy et al., 2007). Short-duration electrostimulation-based strength training has been shown to enhance MVC force via afferent input–mediated modulation of motor system excitability, particularly through cutaneous sensory pathways activated by electrical muscle stimulation (Hortobágyi and Muffuletti, 2011).

Furthermore, verbal encouragement represents a simple yet effective strategy for acutely augmenting MVC force, with demonstrated efficacy in boosting performance during both exercise and MVC testing (Amagliani et al., 2010; Binboğa et al., 2013; McNair et al., 1996). Nevertheless, the precise neural mechanisms underlying the facilitative effects of verbal encouragement remain insufficiently elucidated.

### 3.1 Cognitive intervention

#### 3.1.1 Unconscious goal pursuit

Unconscious goal pursuit has been shown to acutely enhance MVC force by optimizing the functional state of the motor system via increased activity in the reward-related dopaminergic system during both sustained nonfatiguing and fatiguing contractions (Takarada and Nozaki, 2014; Takarada and Nozaki, 2018). Libet et al. (1983) demonstrated that the readiness potential, indexed by EEG changes over the motor cortex, emerged more than 350 ms prior to the participant’s conscious awareness of the decision to act. Moreover, activation of cortical regions such as the precuneus and frontopolar cortex was observed 2–3 s before conscious intention, suggesting that movement selection precedes awareness. Subsequently, the supplementary motor area (SMA) appears to determine the timing of this decision (Soon et al., 2008). These findings provide compelling evidence that voluntary actions may originate unconsciously, with movement selection occurring prior to awareness, and that the perception of intention follows—supporting the view that free will may be, in part, a constructed experience (Hallett, 2007). Collectively, if excitability within an already active M1 can be further elevated prior to conscious awareness of motor intention—specifically before the corollary discharge of the prepared motor signal from the SMA reaches the parietal cortex—MVC force may be elicited without interference from neural or psychological inhibitory constraints outlined in the Introduction. In principle, this approach may unlock latent reserves for muscular force generation, as MVC force is often constrained by supraspinal inhibition, and submaximal recruitment of muscle fibers has been documented in approximately half of individuals, even when explicitly instructed to exert maximal effort (Belanger and McComas, 1981). This concept has been empirically supported by studies employing subliminal priming paradigms.

Evidence indicates that healthy individuals can unconsciously pursue behavioral goals (Aarts et al., 2008). Moreover, coupling these goals with positive reward signals enhances motivation, eliciting robust actions without conscious intent to achieve the primed objective (Aarts et al., 2008). Subliminal priming using action-related words has been shown to modulate motor performance by activating or suppressing motor representations (D’Ostillio and Garraux, 2012; Sumner et al., 2007). These reward signals, triggered by positive stimuli, are processed within the basal ganglia (BG) —particularly the ventral pallidum—which facilitates increased effort and resource allocation to sustain behavior (Pessiglione et al., 2007). Importantly, subliminal reward-goal priming eliminates demand characteristics such as goal desirability, as participants remain unaware of the primed behavioral states during affective shaping, thereby decoupling conscious awareness from the source of positive affect. Positive affect functions as a reward signal that drives behavior (Shizgal, 1997; 1999) and can drive actions outside conscious awareness (Bijleveld et al., 2009; Pessiglione et al., 2007). Thus, these affective-motivational effects likely arise from unconscious associations between motor goal representations and positive affect, directly enhancing physical capacity. This interpretation aligns with prior studies utilizing affective-motivational priming paradigms, in which physical exertion verbs functioned as motor goals (Custers and Aarts, 2010; Takarada and Nozaki, 2014; 2018; 2024), consistent with the proposed mechanism of unconscious goal pursuit. Unconscious goal pursuit comprises two core processes (Custers and Aarts, 2010). First, actions are prepared via perceptual, sensory, and motor systems based on the ideomotor principle. Second, the brain detects positive reward signals within subcortical limbic structures such as the BG, which are implicated in reward processing and effort mobilization (Pessiglione et al., 2007). The integration of these processes is essential for unconscious goal pursuit (Aarts et al., 2008; Takarada and Nozaki, 2014).

Neural activity within the limbic circuit—originating and terminating in the anterior cingulate cortex (ACC) and medial orbitofrontal cortex (OFC)—modulates M1 excitability and the segmented BG–thalamocortical circuit, and is hypothesized to attenuate motor cortical suppression during sustained MVC under conditions of reduced maximal effort. This effect may occur when additional neural input to M1 during sustained force output diminishes motor cortical inhibition. The limbic system, BG, OFC, thalamus, prefrontal and anterior cingulate cortices, along with premotor and supplementary motor areas and M1, form a re-entrant neural circuit functioning as an acceleration system to enhance M1 output against supraspinal fatigue (Dettmers et al., 1996; Korotkov et al., 2005; Liu et al., 2003; 2007; Post et al., 2009). Motivational inputs to this facilitation system increase M1 activity, thereby augmenting motor output to peripheral effectors (Tanaka and Watanabe, 2012). Reward-based priming stimuli are expected to enhance MVC force by optimizing motor system function during sustained fatiguing maximal effort, as previously observed during brief nonfatiguing exertion (Takarada and Nozaki, 2014). Processing of reward stimuli is mediated by limbic structures interconnected with frontal cortical areas, including the nucleus accumbens and ventral striatum, which promote goal pursuit and evaluate outcome value (Sara, 2009). Indeed, barely visible goal-priming with motivational rewards has been shown to reduce the silent period during sustained maximal force production (Takarada and Nozaki, 2024) and increase handgrip MVC force (Takarada and Nozaki, 2014; 2018). This increase may reflect reduced motor cortical inhibition during maximal effort, driven by unconscious goal pursuit and enhanced reward-related dopaminergic activity. These findings suggest that an additional drive modulating M1 excitability may mitigate motor cortical fatigue during sustained MVC. Thus, M1 activity during fatiguing maximal force production is influenced not only by intrinsic M1 properties but also by inputs from the segmented BG-thalamocortical circuit.

The dopaminergic reward system shares functional and anatomical connections with the pupil-related noradrenergic neuromodulatory system (Sara, 2009). Their interaction, along with shared projections to the prefrontal cortex (PFC), is regulated by the locus coeruleus (LC) (Sara, 2009). Noradrenaline released from the LC causes pupil dilation via α2-receptors, making pupil diameter a widely accepted indirect marker of LC activity (Aston-Jones and Cohen, 2005; Joshi et al., 2016; Phillips et al., 2000). Numerous studies have linked pupil dilation under constant luminance to mental effort or cognitive load, across tasks such as paired-associate learning (Colman and Paivio, 1970; Hess and Polt, 1964; Kahneman and Peavler, 1969) and imagery involving abstract or concrete words (Paivio and Simpson, 1966; 1968; Simpson and Paivio, 1968). Pupil size reflects task engagement, enabling fine-grained, temporal analyses of cognitive effort (Kahneman, 1973; Kahneman and Beatty, 1966). Although it remains unclear whether noradrenergic LC neurons exclusively drive pupil dilation, these neurons are consistently active during dilation (Larsen and Waters, 2018).

Ventral tegmental area (VTA) neurons projecting to the nucleus accumbens—central to dopamine release—are not directly by LC stimulation. Nevertheless, LC activation modulates reciprocal connections between noradrenergic and dopaminergic systems, as well as their interactions within the PFC, where neurons project indirectly to the VTA (Sara, 2009). The close functional and anatomical relationship between these catecholaminergic neuromodulatory systems suggests that enhanced dopaminergic activity may potentiate the pupil-linked noradrenergic system, thereby increasing cortical neuronal gain (Aston-Jones et al., 1997; Aston-Jones and Cohen, 2005). This may facilitate unconscious goal pursuit by concurrently preparing motor actions and detecting positive reward signals, while reinforcing reciprocal dopaminergic-noradrenergic interactions in the PFC. The noradrenergic system contributes to goal-pursuit motivation alongside the dopaminergic system (Bouret and Richmond, 2015; Varazzani et al., 2015). While the noradrenergic system promotes effortful behavior and the overcoming of obstacles, the dopaminergic system modulates value-based decision-making. Together, unconscious goal pursuit may potentiate the noradrenergic system, linking to more strongly motivated voluntary motor behavior, potentially via enhanced dopaminergic activation (Takarada and Nozaki, 2017; 2018; 2022b). The motor system state is consequently optimized through this neuromodulatory interplay. Supporting this, barely visible goal priming with motivational rewards has been shown to attenuate silent periods during both transient (Takarada and Nozaki, 2014) and sustained (Takarada and Nozaki, 2024) MVCs, while concurrently enhancing peak handgrip force and eliciting pupillary dilation (Takarada and Nozaki, 2018).

Notably, the enhancement of the pupil-linked neuromodulatory system and the facilitation of voluntary motor output were observed irrespective of whether the goal priming stimuli were consciously perceived or presented below the threshold of awareness (Takarada and Nozaki, 2018). This evidence indicates that motor resource allocation can be initiated through goal-pursuit mechanisms even in the absence of conscious awareness of the priming cues. Accordingly, implicit motivational processes—driven by associative links between physical exertion and positive affect—may serve as underlying mechanisms of motor-goal priming and act as intrinsic reward signals.

#### 3.1.2 Verbal encouragement

Verbal encouragement is widely recognized as a salient factor in motivating both athletes and healthy individuals to achieve maximum performance during exercise and MVC force testing. Evidence suggests that it can acutely enhance MVC force in various populations, including healthy adults (McNair et al., 1996), trained and untrained college-aged women (Amagliani et al., 2010), male students (Jung and Hallbeck, 2004), and elite athletes (Binboğa et al., 2013). However, the underlying neurophysiological mechanisms remain insufficiently understood and warrant further investigation.

### 3.2 Behavioral intervention

Shouting is linked to the potentiation of pupil-linked neuromodulatory system activity and acutely enhances MVC force by augmenting the motor system state through motor commands generated by shouting. A self-generated shout can increase MVC force by enhancing M1 and/or corticospinal tract excitability, accompanied by potentiation of the pupil-linked neuromodulatory system, which mirrors the effects of motivational priming (Takarada and Nozaki, 2021; Takarada and Nozaki, 2022a). Central control of voice production, including shouting, is mediated by two distinct neural pathways: the limbic vocal control pathway, which governs innate, nonverbal, and emotionally driven vocalizations, and the laryngeal motor cortical (LMC) pathway, which regulates fine voluntary control of voice output—such as speech and song, as well as volitional production of innate vocalizations. These pathways are organized hierarchically, extending from primitive structures in the lower brainstem and spinal cord to higher-order regions, including the ACC and LMC (Simonyan and Horwitz, 2011).

In experimental conditions where participants were instructed to shout immediately before handgrip MVC (Takarada and Nozaki, 2021) or after MVC (Takarada and Nozaki, 2024), functionally coordinated activity between the LMC and ACC–periaqueductal gray (PAG) circuits was found to be essential for effective voice initiation and control. The LMC receives input from the supplementary motor area, which prepares vocal motor execution, and from the inferior frontal gyrus, which supports motor planning for vocalization. The ACC contributes to volitional initiation of voice output and modulation of emotional prosody, while the PAG serves as a central integrator within the limbic vocal control circuit, triggering vocal responses and regulating their intensity. Thus, synchronized activity between the ACC–PAG and LMC pathways is critical for volitional shouting.

The LMC maintains reciprocal connections with subcortical structures, including the claustrum, ventral and mediodorsal thalamus, medial parabrachial nucleus, deep mesencephalic nucleus, and LC, as well as motor-related cortical regions such as the ventral and dorsal premotor cortices, M1, and the supplementary motor area. The ACC, along with other cortical and subcortical regions involved in limbic, sensory, motor, cognitive, and arousal functions, projects directly to the PAG (Dujardin and Jürgens, 2005). Brainstem reticular formations, including the LC, ventrolateral medulla (A1), and dorsal pons (Vth), send noradrenergic projections to the PAG. By increasing activity within the LMC and ACC–PAG circuits, shouting may enhance LC neuron firing and induce pupillary dilation.

The LC is the principal source of noradrenergic input to the forebrain, providing regionally specific input to areas critical for higher-order cognitive and affective processing, including the neocortex and hippocampus. The LC–noradrenergic system enhances sensory signal transmission and information processing within sensory circuits, such as stimulus detection and encoding (Berridge et al., 1993; Berridge and Waterhouse, 2003). However, its influence on central motor systems remains poorly characterized. *In vitro* studies have shown that locally applied norepinephrine (NE) increases the excitability of pyramidal tract neurons in the human motor cortex by modulating rheobase and suppressing accommodation (Foehring et al., 1989). Moreover, single oral doses of noradrenergic agents—including the presynaptic α2-antagonist yohimbine (Plewnia et al., 2001), the selective NE reuptake inhibitor reboxetine (Herwig et al., 2002; Plewnia et al., 2002), and the indirect NE agonist methylphenidate (Ilić et al., 2003; Moll et al., 2003)—have been shown to enhance cortical motor excitability.

NE is thought to modulate motor cortex activity through several mechanisms. First, by reducing slow potassium currents and increasing persistent inward sodium currents, NE enhances excitability of large pyramidal neurons in layer V of the rat motor cortex (Foehring et al., 1989). Second, NE presynaptically inhibits GABAergic interneurons, thereby diminishing evoked inhibitory postsynaptic potentials in the rat sensorimotor cortex (Bennett et al., 1997; 1998). Third, because intracortical facilitation is regulated by GABAA and glutamatergic receptors (Ziemann et al., 1996; 1998), NE activates α1-adrenoreceptors on presynaptic glutamatergic terminals in layer V pyramidal cells of the rat prefrontal cortex, increasing the frequency of spontaneous excitatory postsynaptic potentials (Marek and Aghajanian, 1999). Collectively, these actions enhance intracortical facilitation and suppress intracortical inhibition in motor-related areas.

These facilitatory effects of NE on motor cortical neurons contribute to a shortened cortical silent period and acutely increased MVC force (Takarada and Nozaki, 2021; 2022a). Although our experimental design did not allow for direct quantification of intracortical inhibition, silent periods exceeding 100 ms recorded in hand muscles of healthy individuals are commonly interpreted as markers of cortical inhibition (Cantello et al., 1992). The cortical silent period is primarily generated within M1 (Inghilleri et al., 1993), where GABAB-mediated inhibitory circuits are believed to play a central role (Connors et al., 1988; Siebner et al., 1998; Werhahn et al., 1999). Therefore, shouting may enhance motor excitability by attenuating inhibitory processes within M1.

### 3.3 Afferent-driven intervention

Vascular occlusion during voluntary muscular contraction may facilitate MVC force production by enhancing excitability within the contralateral M1 and/or the corticospinal tract. Takarada et al. (2000a), Takarada and Nozaki (2002), Takarada and Nozaki (2004) demonstrated that vascular occlusion induces significant muscular hypertrophy and concomitant strength gains, even when the exercise load is substantially lower than conventional hypertrophic thresholds. These strength-enhancing effects of ischemic resistance exercise have been supported by numerous studies over the past 2 decades. Low-intensity resistance exercise combined with vascular occlusion augments human muscular strength by increasing muscle activation and neural drive, partly through the additional recruitment of fast-twitch fibers triggered by fatigue-induced mechanisms.

Clinically, vascular occlusion applied to the proximal upper arm with a tourniquet elicits a distinct pressure sensation. Somatosensory input is transmitted from the primary somatosensory cortex (S1)—which shares dense anatomical connectivity with M1— via thalamocortical projections (Kaas et al., 1983; Ghosh et al., 1987; Keysers et al., 2010). Neurophysiological studies in humans employing short-latency afferent inhibition have shown that sensory afferent volleys can attenuate M1 excitability through thalamocortical pathways (Tokimura et al., 2000). Additionally, direct cortico-cortical projections from S1 to M1 have been shown to suppress M1 excitability, as evidenced by reduced motor-evoked potential (MEP) amplitude when ipsilateral S1 stimulation precedes M1 stimulation by 1 ms at suprathreshold intensity (Brown et al., 2019). This is consistent with findings that vascular occlusion-induced activation of group III and IV mechanosensitive and metabosensitive afferents not only diminishes spinal motoneuron excitability (Martin, PG et al., 2006) and lowers motor unit discharge rates (Lowe et al., 2023), but also reduces contralateral M1 excitability (Sidhu, et al., 2018).

Based on these neurophysiological alterations, vascular occlusion likely suppresses excitability within M1 and/or corticospinal output at rest, regardless of whether inhibition is mediated via thalamocortical or direct cortico-cortical pathways. Indeed, H-reflex amplitudes in response to median nerve stimulation decrease during vascular occlusion (Takarada and Nozaki, 2025). Similarly, during muscle contraction under vascular occlusion, M1/corticospinal excitability is attenuated, as indicated by prolonged cortical silent periods (Takarada and Nozaki, 2025). These findings suggest a generalized suppression of neural responsiveness across multiple levels of the motor pathway.

Thus, inhibition at the level of M1, the corticospinal tract, and spinal motoneurons may be compensated for by increased excitatory input from motor-related cortical regions, which project via M1 to spinal motoneurons to facilitate additional motor unit recruitment during occlusion. Because both submaximal and maximal force outputs remain unaffected by occlusion (Takarada and Nozaki, 2025), such compensatory cortical drive likely preserves force output despite transient inhibition. This mechanism appears particularly relevant during maximal effort, given prior evidence of unchanged MVC force under occlusion (Takarada et al., 2006; Takarada et al., 2013; Takarada and Nozaki, 2025). Therefore, even a single bout of muscle contraction with vascular occlusion may engage cortical-level compensatory mechanisms, enhance excitatory input to the spinal cord, and ultimately augment MVC force. In other words, cortical compensation for transient inhibition of both spinal motoneurons and corticospinal excitability during vascular occlusion may induce neural adaptations that enhance motor performance.

### 3.4 Pharmacological intervention

Caffeine and pain modulation have been shown to acutely enhance MVC force primarily through acute neural adaptations occurring at the supraspinal and/or cortical levels. Acute caffeine ingestion has been reported to increase MVC force and explosive isometric strength via mechanisms operating within central neural pathways such as M1 and/or the corticospinal tract (Behrens et al., 2015), thereby potentially contributing to improved muscle endurance. However, the current paucity of sex-specific data underscores the need for further investigation in female populations (Bilondi et al., 2024). Taken together, these findings suggest that caffeine’s force-enhancing effects are mediated by acute central adaptations rather than alterations in spinal excitability. Indeed, studies have demonstrated that MEPs and cortically evoked twitches of the vastus lateralis during low-intensity isometric contractions are significantly increased following caffeine ingestion (Kalmar and Cafarelli, 2006), while H-reflex amplitudes remain unaffected both at rest and during contraction (Behrens, et al., 2015; Kalmar and Cafarelli, 1999). These outcomes are most plausibly explained by caffeine’s antagonism of adenosine receptors (Fredholm, et al., 1999). Acting as an adenosine receptor antagonist, caffeine counteracts the tonic inhibitory effects of adenosine within the central nervous system, which include suppression of excitatory neurotransmitter release and neuronal firing rates (Kalmar, 2005). Additionally, caffeine facilitates dopaminergic transmission through postsynaptic modulation of A2a receptors (Ferré et al., 1997; Fredholm, et al., 1999). Collectively, these findings support the interpretation that acute supraspinal and/or cortical adaptations are primarily responsible for caffeine’s ability to enhance muscular force.

In contrast, MVC force can be experimentally attenuated by pain, most likely through central rather than peripheral mechanisms (Graven-Nielsen et al., 2002), and pain also compromises the ability to maintain steady submaximal force output (Arvanitidis et al., 2025). When centrally mediated muscle pain—particularly clinical pain—is alleviated, improvements in MVC force and submaximal force steadiness may be observed (Arvanitidis et al., 2025; Graven-Nielsen et al., 2002). Notably, reductions in MVC force during isometric knee extension are not accompanied by impairments in contractile properties, as assessed by twitch interpolation, confirming that such inhibition is centrally mediated and not attributable to overt muscle damage (Graven-Nielsen et al., 2002). This implies that in rehabilitation and training contexts involving patients with musculoskeletal pain but without peripheral muscle pathology, analgesic interventions may help expand the pain-free force range, thereby facilitating exercise participation.

Centrally mediated force inhibition during muscle pain may involve spatial facilitation between Ib afferents and group III and IV muscle afferents (Schomburg et al., 1999) and/or pain-induced suppression of muscle spindle function (Matre et al., 2002; Svensson et al., 2000). The former mechanism may decrease reflex sensitivity in agonist muscles while increasing sensitivity in antagonist muscles, thereby diminishing MVC force during pain (Graven-Nielsen et al., 1997; Schomburg, 1990; Svensson et al., 1998; Wang et al., 2000). The latter mechanism reduces motoneuron pool excitability, as evidenced by reduced H-reflex amplitudes in humans (Le Pera et al., 2001; Svensson et al., 1998; Wang et al., 1999). Furthermore, TMS studies have demonstrated that experimental muscle pain reduces cortical motoneuron excitability (Le Pera et al., 2001). Accordingly, effective pain management may restore MVC force and enhance the ability to sustain steady submaximal force outputs.

### 3.5 Stimulation-based intervention

Electrical and magnetic stimulation techniques can also be utilized to modulate MVC force. rTMS applied to the contralesional M1, in combination with MVC immediately preceding rehabilitation training, has been shown to enhance corticospinal excitability, thereby improving MVC force in the paretic upper limb during the early post-stroke phase (Pomeroy et al., 2007). This protocol, which pairs rTMS with voluntary muscle activation during rehabilitation, may acutely enhance MVC force not only in healthy individuals (Urbach and Awiszus, 2000)—as demonstrated by increased cortical voluntary activation estimated via the established twitch interpolation technique (Todd et al., 2003), but also in patients following total knee arthroplasty (Urbach et al., 2005). Short-term strength training incorporating electrical stimulation has been shown to facilitate MVC force gains by modulating motor system excitability through cutaneous afferent pathways (Hortobágyi and Maffiuletti, 2011). Notably, electrical stimulation has been reported to activate not only contralateral primary and secondary sensorimotor cortices but also M1, supplementary motor areas, premotor cortex, ipsilateral cerebellum, anterior cingulate cortex, and thalamus (Blickenstorfer et al., 2009; Han et al., 2003; Smith et al., 2003). Furthermore, converging evidence from behavioral assessments, functional MRI, and TMS studies suggests that enhanced somatosensory input may drive cortical reorganization (Kimberley et al., 2004; Nudo et al., 2001). Taken collectively, these findings underscore the potential of electrical stimulation-based strength training to induce neural adaptations via mechanisms of cortical plasticity. Accordingly, it is plausible that somatosensory and/or nociceptive inputs modulate motor cortical excitability, thereby contributing to functional improvements such as increased muscle strength and enhanced coordination (Francis et al., 2009; Kaelin-Lang et al., 2002; Khaslavskaia and Sinkjaer, 2005; Ogino et al., 2002; Ridding et al., 2000; Ridding et al., 2001).

Although recent systematic reviews have highlighted the potential of tDCS to enhance neuromuscular performance, including improvements in maximal and explosive strength when combined with physical training (Liu et al., 2024), its effects on MVC in healthy adults appear to be small and not consistently significant (Alix-Fages et al., 2019). Therefore, while tDCS is discussed in the text as a complementary stimulation-based intervention, it was not included in Table 1, which focuses on interventions with more robust evidence for MVC enhancement.

**TABLE 1:** Summary of MVC-enhancing interventions, neurophysiological mechanisms, functional outcomes, and population-specific applications.

Target population	Intervention/Strategy	Neurophysiological mechanisms	Applications, outcomes, and notes
Spinal cord injury	Paired corticospinal–motor neuronal stimulation (TMS + exercise)	Activates preserved corticospinal pathways; ↑ corticospinal excitability	MVC ↑ 40%–50%; TMS alone ineffective
Stroke survivors	Strength training + task-specific practice (walking, sit-to-stand); vascular occlusion training; low-intensity aerobic exercise + occlusion; passive occlusion	↑ M1 and corticospinal excitability; hypertrophy with low loads; cortical compensation during occlusion	↑ paretic muscle strength; ↑ ADL (balance, gait, stair climbing, feeding); safe (low-intensity protocols)
Joint disease (OA, post-traumatic, TKA)	TMS during MVC (triple-pulse); vascular occlusion + TMS	↑ M1 and corticospinal excitability; compensatory cortical drive despite inhibition	MVC ↑ (≤60 min); limited voluntary activation improvement; promising for degenerative/post-traumatic dysfunction
Multiple sclerosis	Unilateral resistance training (*weaker-limb focus*); vascular occlusion + unilateral training	↑ M1 and corticospinal excitability; ↑ fast-twitch fiber recruitment	MVC explains 20%–30% of lower-limb function; ↓ asymmetry → ↑ mobility; selective hypertrophy in *weaker limb*
Chronic heart failure	Low-intensity aerobic exercise + vascular occlusion	Compensatory cortical drive; favorable BNP modulation	↑ exercise tolerance; ↑ QOL; safe despite ↓ aerobic capacity
Healthy older adults	High-load resistance training (70%–80% MVC) + verbal cues; low-load occlusion training (20%–30% 1RM); pain management (*analgesia, caffeine*)	Recruitment of high-threshold motor units; ↑ fast-twitch fiber activation; ↓ pain perception	↑ walking performance; ↓ fall risk; independence preserved; hypertrophy with low loads; ↑ adherence with pain control
Athletes	Low-load occlusion training (*hypertrophy*); high-load + occlusion (*MVC*); shouting/*grunting*	Low-load + ischemia → metabolic stress (*lactate ↑, mTORC1 activation*); high-load + occlusion → compensatory cortical drive, ↑ fast-twitch recruitment; shouting → LC activation, ↑ M1 excitability	Hypertrophy ≈ high-load training; MVC ↑; performance gains (e.g., tennis serve velocity ↑)

The column ‘Applications, outcomes, and notes’ summarizes functional improvements (e.g., MVC force, ADL, performance), potential applications across rehabilitation and athletic training contexts, and context-specific considerations (e.g., safety, limitations). Abbreviations: MVC, maximal voluntary contraction; TMS, transcranial magnetic stimulation; NMES, neuromuscular electrical stimulation; ADL, activities of daily living; LC, locus coeruleus; BNP, brain natriuretic peptide; M1, primary motor cortex.

## 4 Potential applications of manipulations that improve maximal muscle force

This section explores the various ways in which increasing MVC force may enhance outcomes across diverse settings and proposes appropriate MVC manipulation techniques tailored to each context.

High-intensity strength training, consisting of four sets of four repetitions at approximately 85%–90% of one-repetition maximum (1RM), has been shown to increase muscle strength (MVC force and 1RM) alongside muscle hypertrophy in healthy, physically active older adults (n = 10). This hypertrophic response may be linked to elevated ATP synthesis rates, potentially mediated by a shift toward a more glycolytic muscle phenotype characterized by a higher proportion of fast myosin heavy chain (MHC) isoforms (Berg et al., 2018). Such high-intensity protocols, involving a limited number of repetitions performed at near-maximal effort, have proven effective in enhancing muscle strength across various age groups and sexes (Kittilsen et al., 2021), suggesting that enhancing MVC during high-intensity training may further optimize strength adaptations in a broad population.

However, a substantial proportion of participants (27 out of 76, 11 males and 16 females, aged 21–67 years) were unable to complete the high-intensity (∼85–90% 1RM) resistance training protocol. This dropout rate—approximately 36% of the total cohort (33 men and 43 women, aged 20–76 years)— raises concerns regarding the feasibility of such protocols. Notably, nine of these dropouts were directly attributed to the intervention itself, accounting for approximately 12% of the total sample and 34% of all dropouts. These findings indicate that even individuals with prior experience in strength training may find high-intensity protocols excessively demanding. It is therefore plausible that frail older adults (Marshall-McKenna et al., 2021), patients with joint disorders (Urbach et al., 2005), cardiovascular disease (Paluch et al., 2024), or spinal cord injury (Krogh et al., 2022) may be particularly vulnerable to dropout when exposed to such high-intensity regimens. Accordingly, MVC manipulation strategies should be customized to address the specific needs and limitations of each population.

Importantly, all MVC manipulations should be integrated with motivational goal-priming techniques. The force-potentiating effect may be amplified by affective-motivational influences such as unconscious goal pursuit, age, sex, or clinical status. Unconscious goal pursuit is thought to activate the noradrenergic system and enhance voluntary motor behavior, potentially via dopaminergic system facilitation (Takarada and Nozaki, 2017; 2018; 2022b), thereby inducing a heightened motor system state. Empirical evidence indicates that barely perceptible goal-priming with motivational rewards can reduce the silent period during both transient (Takarada and Nozaki, 2014) and sustained (Takarada and Nozaki, 2024) MVC, accompanied by pupillary dilation (Takarada and Nozaki, 2018).

In practical settings, individuals frequently experience emotional fluctuations during gym-based exercise or rehabilitation. To ensure consistent performance under such variable emotional conditions, current findings suggest that gym instructors, sports trainers, physical therapists, and coaches may enhance outcomes by fostering positive affective states. This may be achieved through strategic goal priming interventions, such as presenting positive verbal cues and behavioral representations—either consciously or unconsciously—during sustained fatiguing voluntary contractions involving maximal force production. Such approaches may encourage individuals to exert maximal effort even when confronting physical limitations, regardless of exercise intensity. Future research should investigate the muscular force-potentiating effects of unconscious goal pursuit across different MVC manipulation paradigms.

### 4.1 Applications for rehabilitation

The effectiveness of MVC force manipulation strategies may vary depending on the specific rehabilitation or training context. For example, interventions that more effectively engage preserved neural pathways are essential for promoting functional recovery in individuals with spinal cord injury. Paired corticospinal–motoneuronal stimulation using TMS, when combined with exercise, appears particularly promising, as this approach can increase MVC force by approximately 40%–50% in individuals with spinal cord injury, whereas TMS alone does not produce comparable improvements (Jo and Perez, 2020).

Stroke typically results in pronounced muscle weakness on the paretic side, with milder deficits observed contralaterally. Restoration, maintenance, and enhancement of muscular function are critical therapeutic objectives for stroke survivors (Eng, 2004). Paretic muscle strength is closely linked to several essential activities of daily living, including balance (Hamrin et al., 1982), ambulation (Kim and Eng, 2003; Nadeau et al., 1999a; 1999b), rising from a seated position (Cameron et al., 2003), stair negotiation, and feeding (Bohannon et al., 1991), all of which are integral to overall quality of life.

Similarly, individuals with chronic heart failure (CHF), who often experience reduced exercise tolerance and diminished quality of life, may benefit from interventions aimed at restoring and preserving muscle function (Tanaka and Takarada, 2018). Joint disorders—including arthritis, traumatic injury, and postoperative conditions—are frequently associated with weakness in muscle groups surrounding the affected joint. This muscle dysfunction is thought to be partially attributable to impaired voluntary activation of the involved musculature (Hurley et al., 1997; Urbach and Awiszus, 2002). Accordingly, strategies that enhance voluntary muscle activation may improve muscle performance and increase MVC force—effects that have been demonstrated in previous studies (Urbach et al., 2005).

#### 4.1.1 Stroke

Strength training is well established to induce neural adaptations that enhance force-generating capacity in individual post-stroke. However, due to stroke-related impairments in motor coordination, task-specific practice is often necessary to translate strength gains into functional improvements. Indeed, numerous task-oriented interventions incorporating progressive resistance or strength training within functional activities—such as treadmill walking, repetitive sit-to-stand movements, and circuit-based training—have demonstrated efficacy in improving functional performance (Dean et al., 2000; Duncan et al., 2003; Eng et al., 2003; Rimmer et al., 2000).

Combining task-specific practice (e.g., sit-to-stand) with external compression applied to the proximal region of the paretic thigh may offer additional benefits, as it facilitates muscle strengthening during functional movement. Vascular occlusion during voluntary contraction has been shown to acutely augment excitability within the contralateral M1 and/or corticospinal tract without inducing neuromuscular failure (Takarada and Nozaki, 2025). Moreover, repeated application of occlusive stimuli in conjunction with low-intensity muscle contractions (20%–50% 1RM) has been associated with hypertrophy of lower limb musculature (Takarada et al., 2002; Takarada et al., 2004).

Importantly, concerns regarding adverse hemodynamic responses—such as excessive elevations in blood pressure (MacDougall et al., 1992) or skeletal muscle injury (McCully, 1986; Roth et al., 2000)—are mitigated by the use of bodyweight-only exercise during occlusion-based functional training. Indeed, low-intensity cycling exercise combined with vascular occlusion has been validated as a safe and effective rehabilitation modality for individuals with CHF. In this population, muscle atrophy-related exercise intolerance often precludes participation in conventional aerobic training, even at low intensities. Occlusion-based aerobic exercise not only improves exercise tolerance but also favorably modulates biomarkers such as serum brain natriuretic peptide (BNP) levels (Tanaka and Takarada, 2018).

Additionally, passive applications of occlusive stimuli—i.e., without accompanying exercise—may serve as a preventive strategy against disuse-induced muscle atrophy in stroke patients during bed rest. This approach may reduce susceptibility to skeletal muscle injury during subsequent high-intensity or unfamiliar exercise bouts (Allen et al., 2004). Such atrophy-attenuating effects have previously been demonstrated following surgical procedures, where periodic occlusion alone preserved knee extensor muscle mass in the absence of active contraction (Takarada et al., 2000b).

#### 4.1.2 Join disease

Joint pathology is known to induce weakness and atrophy in adjacent musculature. Several studies have demonstrated that restoring muscular strength to normative levels remains challenging and, in many cases, unattainable (Hurley et al., 1992; Kannus and Järvinen, 1991). A key contributor to this deficit is the voluntary activation failure in the quadriceps femoris muscle, which not only impairs strength recovery but also limits the efficacy of physiotherapeutic interventions (Hurley et al., 1992; Stokes and Young, 1984). Accordingly, individuals with joint disease may benefit from strategies aimed at enhancing voluntary neuromuscular activation to mitigate muscle dysfunction.

Indeed, three consecutive single-pulse TMS stimuli applied over the motor cortex during MVC in individuals following total knee arthroplasty (mean age: 62 years) were shown to increase sustained MVC force for up to 60 min post-stimulation (Urbach et al., 2005). However, this augmentation in force was not accompanied by a statistically significant improvement in voluntary activation, particularly when compared to TMS administered during muscle relaxation.

The force-enhancing effect of repeated TMS during MVC may be further potentiated by concurrent vascular occlusion, achieved via tourniquet placement at the proximal region of the limb. Tourniquet-induced occlusion is known to transiently suppress spinal motoneuron and corticospinal tract excitability, potentially eliciting compensatory cortical input to M1 and/or corticospinal pathways, thereby amplifying voluntary drive during submaximal exertion. Notably, such cortical compensation may also occur during MVC, even in the absence of measurable changes in voluntary activation. This is supported by findings indicating that MVC force remains unchanged with or without vascular occlusion, despite the presence of motor system inhibition (Takarada and Nozaki, 2025).

Taken together, the combined application of consecutive single-pulse TMS during MVC and vascular occlusion may yield further improvements in MVC force, likely through enhanced excitability of M1 and/or corticospinal tract. This approach may hold therapeutic promise for conditions characterized by muscle weakness secondary to impaired voluntary activation, such as degenerative joint disease (Becker et al., 2004) or post-traumatic joint dysfunction (Hurley, 1999; Urbach and Awiszus, 2002; Urbach et al., 2001).

#### 4.1.3 Multiple sclerosis

MVC force reportedly explains approximately 20%–30% of the variance in lower-limb functional capacity tests (e.g., walking) among individuals with multiple sclerosis, highlighting the importance of enhancing muscular strength to support functional mobility (Ramari et al., 2020). Reducing interlimb strength asymmetry is also essential, as functional performance appears to be more closely linked to the strength of the weaker limb (Ramari et al., 2020).

Targeted unilateral resistance training has been identified as a promising strategy to elicit neuromuscular and strength gains preferentially in the weaker leg (Kjølhede et al., 2015). Furthermore, combining unilateral resistance exercise with vascular occlusion may amplify these adaptations, potentially by facilitating repeated muscular contractions under hypoxic conditions during extended rehabilitation. This method may increase compensatory neural drive to spinal motoneurons via increased excitability of M1 and/or the corticospinal tract (Takarada and Nozaki, 2025), thereby promoting the selective or augmented recruitment of fast-twitch fibers—mechanisms commonly observed in high-intensity resistance training (Takarada et al., 2000a; Takarada et al., 2000c).

### 4.2 Applications for healthy older adults

Healthy older adults may enhance walking performance, reduce fall risk, and enhance overall quality of life by increasing MVC force through greater voluntary activation of lower-limb musculature (Hvid et al., 2016; Marshall-McKenna et al., 2021). Preserving muscle strength throughout the lifespan has been associated with a lower incidence of functional impairments (Brill et al., 2000), thereby supporting successful aging and reducing the likelihood of losing independence (McLeod et al., 2016). Nevertheless, muscle strength typically declines by approximately 24%–36% between the ages of 50 and 70, reflecting normative age-related changes (Evans, 1997).

A minimum threshold of muscular strength, combined with an adequate rate of force development (RFD), is essential for performing activities of daily living. However, efficient force generation depends on precise neuromuscular coordination, which deteriorates with age and contributes to strength loss (Clark and Manini, 2008). At the neurophysiological level, age-related reductions in cortical and spinal excitability (Kido et al., 2004; Scaglioni et al., 2002; Smith et al., 2009), declines in motor unit number and size (McNeil et al., 2005), and alterations in motor unit discharge behavior (Kamen et al., 1995) have been identified as key mechanisms underlying impaired force production. Accordingly, resistance training protocols that stimulate corticospinal tract activity are critical for preserving muscle strength in older adults.

Voluntary muscle activation may be enhanced through a combination of high-intensity loading (70%–80% of MVC force) and explicit verbal instructions encouraging participants to perform the concentric phase of lower-limb contractions as explosively as possible (e.g., “as fast and forcefully as you can”). Compared with low-load training, high-load resistance exercise more effectively recruits large, high-threshold motor units (Hvid et al., 2016; Schoenfeld, et al., 2014). In such contexts, preferential or compensatory recruitment of fast-twitch fibers may also be promoted by augmented neuronal input to spinal motoneurons following vascular occlusion. This response may be mediated by transient suppression of M1 and/or corticospinal transmission, as well as increased excitability of spinal motoneurons (Takarada and Nozaki, 2025). Consequently, fast-twitch fibers may be selectively or additionally activated even during low-load resistance training when combined with vascular occlusion, thereby promoting gains in MVC force and muscle hypertrophy (Takarada et al., 2000c). This approach appears particularly advantageous for frail or sedentary older adults, as low-intensity (20%–30% 1RM) occlusive training can elicit substantial hypertrophic responses while minimizing orthopedic risks associated with high mechanical stress (Hughes et al., 2017; McLeod et al., 2016).

Nonetheless, resistance training to volitional failure in older adults requires caution—not only due to age-related conditions such as osteoarthritis or skin fragility, but also because of the need to manage exercise-induced muscle pain (e.g., during knee extension exercises) (Marshall-McKenna et al., 2021). Approximately 30% of participants have been reported to discontinue training due to pain. Indeed, when centrally mediated muscle pain—particularly clinical pain—is effectively controlled, MVC force may improve and submaximal force output can be sustained (Arvanitidis et al., 2025; Graven-Nielsen et al., 2002). Emerging evidence also indicates that caffeine ingestion may attenuate exercise-related discomfort, enabling individuals to train at higher intensities and/or for longer durations (Moll et al., 2003). Therefore, strategies aimed at reducing muscle pain—such as pre-exercise caffeine intake—may enhance the effectiveness of resistance training in healthy older adults.

### 4.3 Athletic and sporting applications

High mechanical loading of muscle tissue (≥70% of 1RM) has long been regarded as a fundamental stimulus for increases in maximal strength (American College of Sports Medicine, 2009; Kraemer and Ratamess, 2004; Sale et al., 1987; Vierck et al., 2000), as conventional strength training programs utilizing such loads typically result in simultaneous improvement in muscle strength and hypertrophy (Aagaard et al., 2001; Häkkinen et al., 1998). However, the mechanisms underlying muscle hypertrophy—whether via direct mechanotransduction or growth factor-mediated signaling—remain incompletely understood (for a review, see Duchateau et al., 2021). Importantly, similar hypertrophic responses have been reported following both low-load (≤60% 1RM) and high-load (>60% 1RM) resistance training, despite the lack of significant differences in isometric strength outcomes (Schoenfeld et al., 2014).

Meanwhile, accumulating evidence indicates that low-load exercise performed under blood flow restriction can elicit improvements in muscle performance, such as MVC force, comparable to those achieved through high-load resistance training (Duchateau et al., 2021), thereby establishing blood flow restriction as an effective method for enhancing MVC force (Hughes et al., 2017). Given the reduced mechanical stress associated with blood flow restriction, it has been suggested that muscle hypertrophy under these conditions may not be primarily mediated by mechanotransduction. However, alternative mechanisms—particularly those involving metabolic stress induced by blood flow restriction—remain to be clarified, including their role in promoting hypertrophy alongside increased MVC force.

Both conventional high-load resistance training and low-intensity ischemic resistance training have been shown to enhance MVC force and induce hypertrophy, ultimately contributing to improved athletic performance. For example, sprint cyclists benefit from increased MVC force in the lower extremities through conventional strength training, as MVC force directly influences torque magnitude and production rate across pedaling cadence (Cormie et al., 2011; Maffiuletti et al., 2016). MVC force is a primary determinant of sprint cycling performance, underscoring its direct relevance to competitive outcomes (Douglas et al., 2021).

Moreover, significant improvements in knee extension and flexion strength, as well as quadriceps and hamstrings hypertrophy, have been documented following low-load (20%–50%1RM) resistance exercises with vascular occlusion in elite union rugby players (Takarada et al., 2002) and female netball athletes (Manimmanakorn et al., 2013a; Manimmanakorn et al., 2013b), resulting in enhanced performance on sport-specific fitness tests such as the 5 m sprint, 505 agility, and 20 m shuttle run (Manimmanakorn et al., 2013b). Thus, well-trained athletes can achieve performance gains through low-intensity ischemic resistance training comparable to those attained through high-load resistance training.

Given its reduced mechanical demands, ischemic training represents a practical and appealing alternative for enhancing MVC force. Low-load training with vascular occlusion is generally considered safe (Hughes et al., 2017; Manini and Clark, 2009; Wernbom et al., 2020) and is less likely to induce adverse effects associated with high mechanical loading. Accordingly, it may serve as a viable substitute for high-load resistance training in patients (Hughes et al., 2017) and in healthy sedentary or active individuals across age groups (Grønfeldt et al., 2020; Lixandrão et al., 2018; Takarada et al., 2000c; 2004).

The defining feature of resistance exercise with vascular occlusion is its low load. This form of low-load ischemic exercise induces lactate accumulation distal to the occluded site, resulting in increases in muscle CSA comparable to those observed during high-load exercise under normal perfusion (Takarada et al., 2000c). These *in vivo* findings are supported by *in vitro* studies demonstrating lactate’s anabolic properties: in cell culture, lactate enhances myogenesis (Willkomm et al., 2014; Oishi et al., 2015) and increases phosphorylation of p70S6K, a downstream effector of the mammalian target of rapamycin complex 1 (mTORC1) (Oishi et al., 2015). Additionally, rodent models have shown that elevated lactate concentrations modulate muscle differentiation by regulating myogenic protein networks, leading to increased expression of neonatal myosin heavy chain (MHC) and myotube hypertrophy (Tsukamoto et al., 2018). This metabolic stress acts as an anabolic signal (Goto et al., 2005; Nalbandian et al., 2016; Ozaki et al., 2016), particularly under ischemic conditions combined with low-load exercise.

In contrast, vascular occlusion is not sustained during high-load (80% 1RM) resistance exercise with occlusion, likely due to the “pumping” action of strong muscular contractions (Takarada et al., 2000c). These contractions enhance venous outflow (Bonde-Petersen et al., 1975; Mitchell et al., 1980; Walløe and Wesche, 1988), facilitating clearance of metabolites such as lactate, hydrogen ions (H+), inorganic phosphate (Pi), and adenosine diphosphate (ADP) (McMahon et al., 2002). Indeed, no significant differences in plasma lactate concentration or post-exercise hyperemia were observed between high-load exercise with and without occlusion (Takarada et al., 2000c). Consequently, the anabolic effects of metabolic stress—a key driver of adaptation in ischemic resistance training (Schoenfeld, 2013; Schott et al., 1995; Takarada et al., 2000a; 2000b)— are difficult to achieve under high-load conditions. Therefore, vascular occlusion should be paired with low-load resistance exercise when muscle hypertrophy is the primary goal. Notably, bodyweight exercise such as squats, even when combined with low (20%1 RM) external loads and occlusion, has not consistently produced significant hypertrophic outcomes (Luebbers et al., 2014; Yamanaka et al., 2012).

Recent evidence highlights lactate not only as a metabolic by-product but also as a signaling molecule influencing brain excitability. The lactate shuttle theory established its role as a key energy substrate and signaling mediator across tissues (Brooks, 2018), while subsequent work has emphasized its neuromodulatory functions within neuronal networks (Mosienko et al., 2015). A recent scoping review further suggests that elevated lactate levels during high-intensity exercise may modulate corticospinal excitability, although findings remain heterogeneous (Gibson et al., 2024). Together, these studies support the view that lactate accumulation during low-load ischemic or occlusion training could contribute not only to muscle hypertrophy but also to changes in M1 excitability, thereby enhancing MVC force.

In addition, enhanced metabolite levels during blood flow restriction increase neural drive to muscles, leading to greater recruitment of fast-twitch fibers due to fatigue-induced activation (Takarada et al., 2000b; Takarada et al., 2000c). Recent findings indicate that vascular occlusion transiently inhibits both spinal motoneuron and corticospinal tract excitability, resulting in compensatory input to the M1 and/or corticospinal tract from motor-related cortical regions (Takarada and Nozaki, 2025). This cortical compensation may be active during MVC, since MVC force remains unchanged despite vascular occlusion-induced motor system inhibition (Takarada and Nozaki, 2025). Accordingly, high-load resistance exercise combined with vascular occlusion may enhance MVC force via increased M1 and corticospinal tract activity. Given that low-load resistance exercise with vascular occlusion elicits lower motor unit recruitment than high-load exercise without occlusion (Cook et al., 2013; Manini and Clark, 2009), combining vascular occlusion with high-load exercise is recommended. This approach likely promotes preferential and/or additional recruitment of fast-twitch fibers through compensatory neuronal inputs to spinal motoneurons immediately following occlusion.

Finally, the mechanism underlying shout-induced force enhancement offers practical benefits: a conscious, self-generated shout during maximal effort may amplify muscular output, particularly when making a final effort despite limitations. Athletes and coaches may deliberately employ shouting to optimize force production and performance. For example, grunting has been shown to significantly increase ball velocity during tennis serves and forehands among collegiate players, accompanied by increased isometric force and peak muscle activation (O’Connell et al., 2014). Thus, shouting may serve as a simple, non-invasive, and cost-effective technique to enhance athletic performance.

**FIGURE 1:**
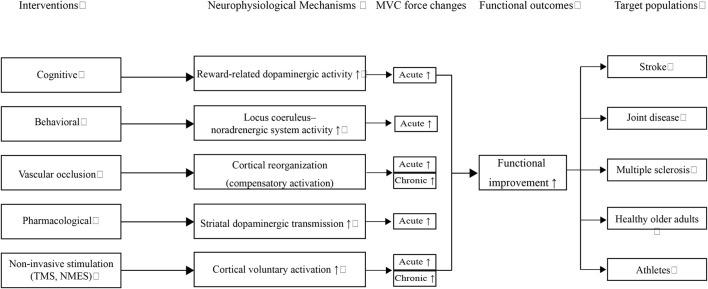
Conceptual flowchart illustrating how diverse interventions (cognitive, behavioral, vascular occlusion, pharmacological, and non-invasive stimulation) modulate specific neurophysiological mechanisms, leading to acute and chronic increases in maximal voluntary contraction (MVC) and subsequent functional outcomes. These adaptations benefit multiple target populations, including individuals with stroke, joint disease, multiple sclerosis, healthy older adults, and athletes. Upward arrows (↑) denote enhancement or facilitation of the respective processes.

To integrate these findings into a broader perspective, I propose a conceptual framework (Figure 1) that links diverse interventions to their underlying neurophysiological mechanisms, subsequent MVC force adaptations, and functional outcomes across target populations. Complementary to this framework, Table 1 summarizes representative interventions and their population-specific applications, thereby providing concrete examples that support the conceptual model.

## 5 Future directions

This section highlights the necessity for continued research to address existing gaps in the literature regarding strategies to enhance MVC force and, consequently, improve functional outcomes. Although the specific neural mechanisms underlying MVC modulation remain incompletely understood, evidence from human studies indicates that facilitating excitability within M1 and/or the corticospinal tract plays a pivotal role in augmenting MVC force. Three principal neural mechanisms proposed to mediate MVC enhancement through cognitive, behavioral, and afferent-driven interventions include: (1) enhanced activity within the reward-associated dopaminergic system, (2) activation of the pupil-linked neuromodulatory system, and (3) heightened sensory input. The development of novel techniques for MVC modulation therefore aims to identify which cognitive, behavioral, or afferent-driven strategies most effectively stimulate these neural pathways.

For example, rTMS applied over the contralateral M1 in combination with MVCs—an approach previously shown to enhance force output—may, when combined with blood flow restriction, further potentiate M1 and/or the corticospinal tract excitability, thereby amplifying MVC force. This potentiation is attributable to the ability of vascular occlusion to enhance M1 and/or corticospinal tract excitability during MVC. In line with this neurophysiological mechanism, integrating self-generated shouting during MVC with vascular occlusion may synergistically elevate M1 and/or corticospinal tract excitability, resulting in greater force output. This effect is likely due to the additional neural activation within M1 elicited by shouting, which intensifies corticospinal activation.

Moreover, caffeine ingestion prior to ischemic muscle contractions may further enhance M1 and/or corticospinal tract excitability, leading to increased MVC force and cortical voluntary activation. Ischemic/hypoxic conditions induced by low-load occlusion contractions or training protocols reduce oxygen availability to the active musculature, resulting in the accumulation of metabolic by-products such as lactate, H+, Pi, ADP, and others. Conversely, adenosine promotes the release of histamine and serotonin from mast cells, contributing to pain and edema via A_3_ receptor activation (Sawynok et al., 1997). Its pronociceptive effects have also been linked to elevated cyclic AMP (cAMP) levels in sensory nerve terminals (Taiwo et al., 1991). As an adenosine receptor antagonist, caffeine may attenuate nociceptor activation and peripheral pain signaling during ischemic muscular occlusion, thereby reinforcing MVC force.

In addition, combining the aforementioned MVC-enhancing techniques with motivational goal priming may further amplify MVC force through the affective-motivational effects of unconscious goal pursuit. This mechanism may activate the noradrenergic system and elevate voluntary motor drive, potentially via dopaminergic system potentiation (Takarada and Nozaki, 2017; Takarada et al., 2018; Takarada et al., 2024), thereby amplifying motor system excitability. Subliminal goal priming with motivational rewards has been shown to shorten the silent period during both transient (Takarada and Nozaki, 2014) and sustained (Takarada and Nozaki, 2024) maximal force exertion, and to increase maximal-effort handgrip force, accompanied by pupillary dilation (Takarada and Nozaki, 2018).

In applied contexts, individuals frequently experience emotional fluctuations during gym-based training or rehabilitation. To ensure consistent performance under such variable emotional conditions, the present findings suggest that gym instructors, sports coaches, and physical therapists may optimize the training environment by eliciting positive affective responses through the use of positive adjectives paired with action-related words, delivered either consciously or subliminally. This approach may leverage the force-enhancing effects of unconscious goal pursuit during sustained fatiguing voluntary contractions, including maximal force exertion. Such strategies may enable individuals to maintain maximal effort even when approaching their perceived physical limits, regardless of exercise intensity. Future research should investigate the impact of unconscious goal pursuit on the force-enhancing efficacy of various MVC modulation techniques.

Finally, although verbal encouragement is widely acknowledged to enhance MVC force, the underlying neural mechanisms remain poorly understood. Accordingly, future studies should aim to elucidate these mechanisms from the perspective of motivational cues, defined here as verbal interventions intended to enhance volitional effort during maximal contractions, as the optimal content and delivery modalities of verbal encouragement may vary across populations.

## 6 Conclusion

Maximal voluntary contraction (MVC) force can be modulated through diverse interventions involving distinct neural pathways. Enhancing excitability within the M1 and/or the corticospinal tract appears critical for optimizing MVC output in humans. These approaches hold promise for application in both rehabilitation and athletic performance contexts. Further investigation into the neurophysiological mechanisms underlying MVC enhancement is warranted to inform targeted strategies for clinical and sport-specific outcomes.
